# Supplementary material for: The Impact of Neoadjuvant Chemotherapy on Survival Outcomes in Gastric Signet-Ring Cell Carcinoma: An International Multicenter Study
Source: Cancers (Basel). 2025 Jul 22;17(15):2419. doi: 10.3390/cancers17152419 (PMC12345905; doi:10.3390/cancers17152419)
Supplement: Supplementary file 1 [file cancers-17-02419-s001.zip › cancers-3585794-supplementary.pdf]

Table S1. Details of NAC regimens.

NAC regimens	N=292 (%)	Duration (cycles)
S-1 (S), Oxaliplatin (OX) (SOX)	170 (58.2)	5.2±2.1
Docetaxel(D), Oxaliplatin(O) and S-1(S) (DOS)	77 (26.3）	3.1±3.8
Capecitabine (CAPE), oxaliplatin (OX) (CAPEOX)	30 (10.2)	4.2±0.9
5-FU, Oxaliplatin (OX), Leucovorin (FOLFOX)	6 (2.1)	3.0±1.1
5-FU (F), Oxaliplatin (O), leucovorin (L), Docetaxel (T) (FLOT)	9 (3.1)	4.5±2.2

Table S2. Details of adjuvant therapy.

Variables	SEER database (n=1773, %)		NCC database (n=1289, %)		Total (n=3062, %)	
	NAC (n=436)	Upfront surgery (n=1337)	NAC (n=292)	Upfront surgery (n=997)	NAC (n=728)	Upfront surgery (n=2334)
Adjuvant treatment						
Chemotherapy	107	189	152	304	259	493
Chemoradiotherapy	40	348	-	-	40	348
Radiotherapy alone	33	21	-	-	33	21
None	256	779	140	693	396	1472

Table S3. Patterns of Recurrence in the NCC database.

	NAC (n=292)	Upfront surgery group (n=997)	*P*
**Local recurrence**	39 (65.0%)	166 (67.8%)	0.68
-Curative-intent surgery	25	120	0.31
**Distant metastssis**	21 (35.0%)	79 (32.2%)	0.67
Bone	1	4	0.65
Peritoneal	7	45	0.28
Central pevis	1	4	0.65
Liver	9	15	0.10
Lung	3	11	0.41
- Systemic chemotherapy*	13	50	0.82
- Radiotherapy	2	5	0.45
Total (%)	60 (20.5)	245 (24.5)	0.16

*Common regimens: SOX, single-agent 5-fluorouracil, or multiagent chemotherapy (a combination of 5-fluorouracil and cisplatin/oxaliplatin, doxorubicin, or paclitaxel/docetaxel)

Table S4. R packages utilized in this study.

R packages	
survival (v3.1-8)	For Kaplan-Meier survival curves (survfit), log-rank tests (survdiff), and Cox proportional hazards models (coxph).
survminer (v0.4.9)	To visualize survival curves and generate publication-quality plots.
dplyr (v0.7.6) and tidyr (v0.8.3)	For data preprocessing, filtering, and merging SEER/NCC datasets.
ggplot2 (v3.3.5)	For exploratory data visualization.
Stats (v3.6.2)	For Statistical tests
